# Evaluating Medication Habits in Medical Mission Patients: A Cross-Sectional Study

**DOI:** 10.7759/cureus.10533

**Published:** 2020-09-18

**Authors:** Jessica H Rehrig, Lucia Vitale, Divya L Padmanabhan, Sharmeen Jaffry, Jane Suh, AnneMarie Tomosky, Christopher Boni

**Affiliations:** 1 Osteopathic Medicine, University of New England, Biddeford, USA; 2 Neurology, North Shore University Hospital, Long Island, USA; 3 Politics Department, University of California, Santa Cruz, USA; 4 College of Science & Technology, Temple University, Philadelphia, USA; 5 Internal Medicine, Waves of Health, Rutherford, USA

**Keywords:** short term medical missions, international primary care, low income countries, public health, global volunteering

## Abstract

Background

Short-term medical missions (STMMs) are a highly debated and largely understudied form of international volunteer work. With growing dedication to health care abroad, research evaluating their impact is crucial to ensure continued interventions are effective in improving medical care. STMM care varies in length, frequency, size, location, services offered, and country of origin and destination. This makes systematic evaluation of STMMs difficult. In addition, the transient nature of patient visits makes trending STMM’s impact on long-term health outcomes complex.

Despite intermittent availability, primary care missions offering pharmaceutical supplies have the unique opportunity to provide continued care to the community via free prescription supplies each visit. Given the challenges with measuring long-term outcomes in this population, it is unknown if these donated medications have any impact on patient health outcomes. As medication noncompliance is known to hinder health outcomes, our study chose to evaluate patient medication habits to see if these prescription supplies were being utilized appropriately. To our knowledge, no study has surveyed medical mission patients to explore their access and utilization of medication.

Methods

A cross-sectional study was conducted using a patient survey to identify risks and/or factors associated with medication noncompliance in patients visiting the medical mission, Waves of Health (WOH). For over 10 years, WOH has organized biannual seven-day trips to the Dominican Republic. The multi-question survey was translated into the native language, Spanish with Dominican dialect. Noncompliance was defined through the survey question “Did you run out of your prescription medication at all during the past year?” Spanish speaking participants, of both sexes and age ≥18 years old, who visited the mission clinic in November 2019 met inclusion for this study. Patients from Haiti or age <18 years old were excluded. Participation was voluntary.

Survey items were dichotomized for univariate analysis to identify factors associated with running out of medication. To explore predictors of running out of medicine, we performed multivariate logistic regression analysis by ENTER method.

Results

Of 127 patients, over half (58.3% [74]) reported running out of medication. Inadequate access to healthcare, daily medication use, and rationing personal medications were all significantly associated with running out of medicine. Frequency of WOH visits was not associated with running out of medication. Multivariate regression showed that being on daily medication and rationing personal medications were statistically significant predictors of running out of medicines. Access to healthcare, frequency of WOH visits, and WOH medication supply were not predictors of running out of medication.

Conclusion

Mission interventions to improve medication practices should be explored due to the high number of patients who reported improper utilization of medication. In order to improve health outcomes in primary care settings, patients must play an active role in their care and understand the importance of taking their medication as prescribed for optimal disease management. Primary care STMMs may relieve short-term health concerns, but without proper utilization of chronic disease medications, it is unclear if STMMs role is impactful in long-term health outcomes.

## Introduction

Short-term medical missions (STMMs) are a popular and highly debated form of volunteer work amongst healthcare providers [1,2]. Defined as a trip providing health care to an underserved community for less than eight weeks at a time, STMMs differ in visit frequency, size, and services provided. These missions are colloquially sent from high-income to middle- and low-income countries with the goal of improving health outcomes in these regions. Each year, the United States alone sends roughly 6000 missions and 250 million dollars to surgical, medical, and pharmaceutical care abroad [1].

Despite growing dedication to missions, empirical data supporting antidotal-reports of improved health outcomes abroad, especially in the setting of primary care, is sparse and stagnant [2,3]. Amongst other reasons for this gap in academia, quantitative research is difficult to conduct in the medical mission setting. The transient nature of patient visits combined with STMM’s limited resources and clinic availability makes research on long-term health outcomes complex [4]. Nonetheless, finding means to statistically access health outcomes is critical, as mission work revolves around their improvement. Given the above challenges, our study chose first to identify a factor in the medical mission population that could deter health improvements despite mission intervention. As medication noncompliance is known to hinder health outcomes, we evaluated patient medication habits it is important to be aware of population, mission personnel could implement changes with measurable outcomes to address these concerns [5,6]. Data from these interventions simultaneously would improve our understanding of the role medical missions play in middle- and low-income communities. 

Our study aimed to assess medication noncompliance in patients visiting Waves of Health (WOH), a nonprofit short-term medical mission from the United States that organizes primary care visits in the Dominican Republic [7]. For over 10 years, WOH has organized two seven-day trips a year to four clinical sites in the Dominican Republic: Barrio Sur, La Vigìa, Sabana Larga, and Campo de Aviaciòn. By exploring associations between medication noncompliance and other health habits, our research held implications for improving STMM medication management strategies. Related health practices and factors were explored for future research opportunity. To our knowledge, no study has surveyed STMM patients to explore their medication habits.

## Materials and methods

We conducted a cross-sectional study to identify risks and/or factors associated with medication noncompliance in the WOH medical mission population. Participants were patients visiting the medical mission, WOH, during the 2019 Fall mission. Our target was to survey 10%, or 115 patients, of the visiting population during the November 2019 mission. Surveys were conducted every day on this seven-day trip. Spanish speaking participants, of both sexes and age ≥18 years old, who visited the mission clinic in November 2019 met inclusion for this study. Patients who spoke Creole as their native language or age <18 years old were excluded. 

During pilot survey data collection in April 2019 and November 2018, we attempted to utilize standardized instruments to evaluate medication adherence but were met by challenges. Many patients were illiterate, requiring help with a translator, and unfamiliar phrasing or terminology were concerns for inaccurate patient answers. Therefore, for this study we utilized literature review and standardized instruments in the setting of qualitative insights from local providers to create survey items appropriate for our patient population. 

The main survey item we used to access medication noncompliance was, "Did you run out of your prescription medication at all during the past year?” As WOH supplies patients with free medication stores to continue their regimen until the next mission trip, if patients come regularly to the mission they should, in theory, not run out of medication. Also, in the Dominican Republic, patients do not require a prescription from a provider, as medications are available over the counter. Therefore, even patients who do not have access to a physician could have access to medication in their community if available and financially affordable. Therefore, this was felt to be the most appropriate survey item to assess medication adherence as prior pilot questions asking patients if they took medication as prescribed yielded biased results. Our survey also asked patients if they took medication daily, if they rationed personal medication over the last year, and if WOH had all the medication they needed. If patients reported running out or rationing medication, they were asked if this was due to access, financial concerns, sharing with others, or another reason. Additionally, patients were asked how many times they visited WOH, and if they were able to access healthcare over the past year. This was to access if there was a correlation between healthcare status and medication adherence. We allowed room for elaboration on responses, but questions were made to be direct to limit difficulties with translation.

Research persons were situated outside clinic rooms near the common exit and approached every 10th patient to exit to participate in the study. Patients were made aware surveys were anonymous and their answers would help identify strategies to improve mission care. All patients were made aware of their right to decline participation at any time. If they agreed to participate, the survey could either be completed alone or with the help of the research translator. The survey was written in the local language, Spanish, and dialect, Dominican, but could also be vocalized by mission interpreters for the many patients who were not comfortable reading in their native language. 

The Institutional Review Board (IRB) at the University of New England College of Osteopathic Medicine waived the requirement of ethical clearance for this study. Before orally conducting the survey, we provided an oral description of the study and participants’ rights to decline altogether or leave questions unanswered. Oral informed consent was taken from participants before enrolling in this study. To preserve participants’ anonymity, the questionnaire did not incorporate name, address, or signature. Participants did not receive any incentives or financial compensation to participate in the study.

As most questionnaire responses were able to be dichotomized, this allowed statistical analysis to be utilized. All statistical analyses were performed using Statistical Package for the Social Sciences (SPSS) v23 (IBM Corp, Armonk, NY, USA). Results for categorical data were presented in the form of frequency and percentages. The following variable was further explored as a dependent variable: running out of medication. This analysis was completed to assess the association between independent and dependent variables using χ2 test. Association was presented in the form of odds ratio (OR) with 95% confidence interval (CI). Statistical significance was accepted at P < 0.05. Additionally, to explore predictors of running out of medicine we performed multivariate logistic regression analysis by ENTER method. 

## Results

Survey patient characteristics

Our study sample comprised 10% (127) of the 1267 patients treated by the November 2019 Waves of Health mission trip. Of respondents, 77.2% (98) were female and 22.8% (29) were male; there was one survey where sex was not recorded.

Patient survey responses

A visual depiction of our questionnaire data can be found in Table 1. Of the 127 patients interviewed, access to healthcare in the community over the past year was insufficient for 93 (73.2%) patients; the other 34 (26.8%) had been able to see a doctor if they needed. Forty-two (33.1%) had visited WOH clinics >five times and 85 (66.9%) had visited ≤five times. Most patients visited the WOH clinic during this survey for mediation refills (63 [49.6%]) while 60 (47.2%) had a new health concern, and four (3.1%) had come for other reasons, such as to accompany family. The majority of patients reported the mission had all the medication they needed (106 [83.5%]) while 21 (16.5%) left without supplies they needed. Over the past year, 74 (58.3%) patients reported running out of medication; 63 (85.1%) stated it was due to cost and 11 (14.9%) stated it was due to access. There were 71 (55.9%) reports of rationing personal medications: 36 (28.3%) did so to make medication stores last longer, 14 (11%) shared, and 21 (16.5%) had a different reason to ration. Reason to run out of medication and reason to ration medication were only asked to those patients who reported these medication habits over the past year. Medications were taken daily by 73 (57.5%) patients and not daily by 54 (42.5%).

**Table 1:** Survey responses from mission patient sample Reason to run out of medication and reason to ration medication were only asked of those patients who reported these medication habits over the past year.

Characteristics		Sample N (%)
Access to healthcare / year	Lack care	93 (73.2)
	Adequate	34 (26.8)
Total visits to mission	>5	42 (33.1)
	≤ 5	85 (66.9)
Reason to Visit	New health concern	60 (47.2)
	Medication Refill	63 (49.6)
	Other (bring family, etc)	4 (3.1)
Mission medication options	Everything I needed	106 (83.5)
	Did not have everything I needed	21 (16.5)
Frequency of medication	Daily	73 (57.5)
	Not Daily	54 (42.5)
Access to medication / year	Ran Out	74 (58.3)
	Adequate	53 (41.7)
Reason to run out of medication	Money	63 (49.6)
	No Access	11 (8.7)
Ration personal medication / year	Yes	71 (55.9)
	No	56 (44.1)
Reason to ration medication	Make Last Longer	36 (28.3)
	Share	14 (11.0)
	Other	21 (16.5)

Primary Outcome: Factors Associated With Running Out of Medications

Roughly half (58.3% [74]) of our surveyed population reported running out of medication. Univariate analysis was performed to identify any factors associated with this variable. Inadequate access to healthcare (OR: 3.67; 95% CI: 1.67-8.37; P=0.002), daily medication use (OR: 2.72; 95% CI: 1.31-5.64; P=0.007), and rationing personal medications (OR: 6.69; 95% CI: 3.05-14.7; P=0.000) were all significantly associated with running out of medicine. Frequency of WOH visits was not associated with running out of medication. Analysis of factors associated with medication access are displayed in Table 2.

**Table 2:** Factors associated with running out of medications C.I.: confidence interval

Study variables		Run Out N (%)	Adequate N (%)	Odds Ratio	95% C.I.	P value
Access to healthcare	Lack care	62 (83.8)	31 (58.5)	3.67	1.61-8.37	0.002
	Adequate	12 (16.2)	22 (41.5)	1	Reference	
Total visits to mission	>5	47 (63.5)	38 (71.7)	0.69	0.32-1.47	0.334
	≤ 5	27 (36.5)	15 (28.3)	1	Reference	
Frequency of medication	Daily	50 (67.6)	23 (43.4)	2.72	1.31-5.64	0.007
	Not daily	24 (32.4)	30 (56.6)	1	Reference	
Ration personal medication	Yes	55 (74.3)	16 (30.2)	6.69	3.05-14.7	0.000
	No	19 (25.7)	37 (69.8)	1	Reference	

Secondary Outcome: Predictors of Running Out of Medicine

To explore predictors of running out of medicine in this population, we performed multivariate logistic regression analysis by ENTER method. This multivariate regression shows that being on daily medication and rationing personal medications are statistically significant predictors of running out of medicines. Access to healthcare, frequency of WOH visits, and WOH medication supply were not predictors of running out of medication. Results of this multivariate analysis can be found in Table 3. 

**Table 3:** Predictors of medication noncompliance WOH: Waves of Health

Variables	Sig.	Exp(B)	95% C.I.for EXP(B)	
			Lower	Upper
Access to healthcare	.071	.413	.158	1.080
Frequency of WOH visits	.474	1.439	.531	3.896
Take daily medication	.031	.378	.156	.917
Ration personal medications	.000	.157	.066	.376
WOH medication supply	.514	.632	.662	1

## Discussion

At WOH, patients are given a free six-month supply of personal medication each visit. They also do not need a prescription to get medication in their country, as medications are available to purchase over the counter, when available, if they know what they take. Despite this, a large portion of patients in our study reported medication habits that are inconsistent with those known to improve health outcomes. Given the prevalence of patients running out or rationing medications, mission efforts to improve access to medication may be deterred by improper patient utilization. Inconsistent prescription use is not unique to patients of lower income countries [8]. Limited access to medical care over the past year was associated with running out of medication, however it was not a significant risk factor. Frequency of patient visits to the medical mission also had no bearing on running out of medication. Therefore, improving access to health resources does not necessarily translate to improved health outcomes via resource utilization.

Patient health education universally is crucial, but unique complexities exist around this activity in a medical mission clinic. Patients have no contact with the mission in-between visits and many are unable to read Spanish prescription instructions, so providers must be confident patients understand how to utilize medication properly before they leave the clinic. Most mission providers require a translator to communicate with their patients. While language barriers have been shown to have detrimental effects on patient care, current research is exploring the role of translation services in various healthcare settings [9]. Interventions should be directed towards further understanding patient medication habits and stressing the importance of taking medications as prescribed.

As health repercussions vary depending on prescription type and dose, it is worth noting the mission mitigates associated risks by providing only select prescriptions. Many anti-infectious agents were one-time doses, to minimize resistance, and insulin, an easy source for accidental hypoglycemia, was not prescribed by visiting mission physicians. Given the number of patients on daily medications, and the prevalence of patients rationing, improving adherence is an efficient modality for medical missions to improve health outcomes. Future research exploring mission prescribing practices, community health literacy and adherence, and barriers to adherence will likely provide strategies for increasing proper utilization of medication amongst a population already predisposed to inadequate healthcare [10].

Limitations

This study is innately limited by its design, which allows only for association of factors and does not suggest causal relationships. The questionnaire was created and conducted orally by medical volunteers, therefore the cultural dialect and language barrier must be addressed. Additionally, our study only interviewed adults, therefore our findings do not reflect the 46.3% of patients under the age of 18 who were not included in the study. 

## Conclusions

We surveyed 10%, or 127 patients, visiting the November 2019 Waves of Health medical mission clinic in the Dominican Republic. Of the surveyed population, 57.5% took daily medications and 106 reported the mission had all the medication they needed. The most common reason to visit the mission, reported by 49.6%, was medication refills. Despite this, 58.3% of patients reported they ran out of medication over the past year, most commonly due to cost. Rationing medication was also highly reported, although reasons for this activity were not conclusive. Inadequate access to healthcare, daily medication use, and rationing personal medications were all significantly associated with running out of medicine while daily medication use and rationing personal medications were statistically significant predictors of running out of medication. Given a large component of medical mission efforts go to improving prescription supplies for their patients, it is important to ensure proper adherence to medication regimens. STMMs should consider interventions to improve medication practices within their populations, as improper utilization of prescriptions will deter mission efforts to manage chronic disease.
